# Metabolomic signatures associated with weight gain and psychosis spectrum diagnoses: A pilot study

**DOI:** 10.3389/fpsyt.2023.1169787

**Published:** 2023-04-24

**Authors:** Jiwon Lee, Kenya Costa-Dookhan, Kristoffer Panganiban, Nicole MacKenzie, Quinn Casuccio Treen, Araba Chintoh, Gary Remington, Daniel J. Müller, Sanjeev Sockalingam, Philip Gerretsen, Marcos Sanches, Alla Karnovsky, Kathleen A. Stringer, Vicki L. Ellingrod, Ivy F. Tso, Stephan F. Taylor, Sri Mahavir Agarwal, Margaret K. Hahn, Kristen M. Ward

**Affiliations:** ^1^Schizophrenia Division, Centre for Addiction and Mental Health, Toronto, ON, Canada; ^2^Institute of Medical Science, University of Toronto, Toronto, ON, Canada; ^3^Department of Psychiatry, University of Toronto, Toronto, ON, Canada; ^4^Department of Pharmacology and Toxicology, University of Toronto, Toronto, ON, Canada; ^5^Pharmacogenetics Research Clinic, Centre for Addiction and Mental Health, Toronto, ON, Canada; ^6^Education, Centre for Addiction and Mental Health, Toronto, ON, Canada; ^7^Geriatric Mental Health Services, Centre for Addiction and Mental Health, Toronto, ON, Canada; ^8^Biostatistics, Centre for Addiction and Mental Health, Toronto, ON, Canada; ^9^Department of Computational Medicine and Bioinformatics, University of Michigan Medical School, Ann Arbor, MI, United States; ^10^Department of Clinical Pharmacy, University of Michigan College of Pharmacy, Ann Arbor, MI, United States; ^11^Department of Psychiatry, University of Michigan Medical School, Ann Arbor, MI, United States; ^12^Department of Psychiatry & Behavioral Health, Ohio State University, Columbus, OH, United States; ^13^Banting and Best Diabetes Centre, University of Toronto, Toronto, ON, Canada

**Keywords:** metabolomics, fatty acids, antipsychotics, schizophrenia, weight gain, psychosis

## Abstract

Psychosis spectrum disorders (PSDs), as well as other severe mental illnesses where psychotic features may be present, like bipolar disorder, are associated with intrinsic metabolic abnormalities. Antipsychotics (APs), the cornerstone of treatment for PSDs, incur additional metabolic adversities including weight gain. Currently, major gaps exist in understanding psychosis illness biomarkers, as well as risk factors and mechanisms for AP-induced weight gain. Metabolomic profiles may identify biomarkers and provide insight into the mechanistic underpinnings of PSDs and antipsychotic-induced weight gain. In this 12-week prospective naturalistic study, we compared serum metabolomic profiles of 25 cases within approximately 1 week of starting an AP to 6 healthy controls at baseline to examine biomarkers of intrinsic metabolic dysfunction in PSDs. In 17 of the case participants with baseline and week 12 samples, we then examined changes in metabolomic profiles over 12 weeks of AP treatment to identify metabolites that may associate with AP-induced weight gain. In the cohort with pre-post data (*n* = 17), we also compared baseline metabolomes of participants who gained ≥5% baseline body weight to those who gained <5% to identify potential biomarkers of antipsychotic-induced weight gain. Minimally AP-exposed cases were distinguished from controls by six fatty acids when compared at baseline, namely reduced levels of palmitoleic acid, lauric acid, and heneicosylic acid, as well as elevated levels of behenic acid, arachidonic acid, and myristoleic acid (FDR < 0.05). Baseline levels of the fatty acid adrenic acid was increased in 11 individuals who experienced a clinically significant body weight gain (≥5%) following 12 weeks of AP exposure as compared to those who did not (FDR = 0.0408). Fatty acids may represent illness biomarkers of PSDs and early predictors of AP-induced weight gain. The findings may hold important clinical implications for early identification of individuals who could benefit from prevention strategies to reduce future cardiometabolic risk, and may lead to novel, targeted treatments to counteract metabolic dysfunction in PSDs.

## 1. Introduction

Patients with a psychosis spectrum disorder (PSD) and patients with severe mental illnesses that present with psychotic features, often develop metabolic comorbidities like obesity, type 2 diabetes, and dyslipidemia, leading to a 2-fold increase in cardiovascular mortality rates as compared to the general population (1, 2). While various factors contribute to the metabolic risk in PSDs, antipsychotics (APs), the cornerstone of treatment for many PSDs, worsen elevated baseline risk through metabolic side effects such as weight gain (3–5). Notably, young patients without prior AP exposure (i.e., AP-naïve) are especially susceptible to early AP- induced weight gain (3, 6), although there is individual variability in the propensity to develop AP-induced weight gain that is not fully understood. Overall, there is a need to identify biomarkers and mechanisms of intrinsic and early AP-induced metabolic dysfunction to guide early targeted metabolic treatment strategies in PSD.

In addition to the effects of APs, metabolic dysfunction has been suggested to be intrinsic to the illness of PSDs. For instance, AP-naïve first-episode psychosis patients demonstrate higher prevalence of metabolic syndrome, elevated triglyceride levels, markers of glucose dysregulation, and increased risk for type 2 diabetes as compared to healthy controls, even prior to receiving AP treatment (7–11). The presence of metabolic abnormalities in AP-naïve FEP patients, who are minimally affected by two major confounders, APs and illness duration, is suggestive of an intrinsic metabolic risk that is conferred by the illness of PSDs. However, the mechanisms underlying intrinsic metabolic dysfunction are understudied. Taken together, there is a need to examine AP-naïve patients to identify biomarkers, predictors, and mechanisms of intrinsic and early AP-induced metabolic dysfunction, which would have important implications for guiding early treatment strategies for individuals with PSDs and developing novel, targeted treatments for metabolic dysfunction in PSDs.

Metabolomics represents a novel tool with potential to shed light on metabolic dysfunction in PSD by characterizing global metabolite profiles. For example, metabolomic studies have identified that higher baseline concentrations, and change over time, of the lipid metabolite lysophosphatidylcholine 14:0 were associated with weight gain following olanzapine treatment in first-episode schizophrenia (12). Nonetheless, few studies have examined intrinsic illness markers of metabolic dysregulation and predictors of AP-induced weight gain in patients with minimal-to-no previous exposure.

The objectives of the current pilot study were to: (1) examine whether minimally AP-exposed cases and controls present with different metabolomic profiles that may represent illness biomarkers not confounded by long-term AP use; (2) identify predictors of AP-induced weight gain by comparing baseline metabolomes between minimally AP-exposed cases who do and do not develop clinically significant body weight (≥5%) following 12 weeks of AP exposure; and (3) examine associations between changes in the metabolome and body weight over 12 weeks of AP exposure.

## 2. Materials and methods

### 2.1. Participants and study design

Participant data was gathered from three independent observational studies, where recruitment has been completed for two case-only studies [(ClinicalTrials.gov ID: NCT02744313. ClinicalTrials.gov ID is not available for the other study) and is ongoing for the remaining case-control study (ClinicalTrials.gov ID: NCT03414151)]. The study protocols were approved by the Research Ethics Board of the Centre for Addiction and Mental Health (030/2017 and 060/2014) and University of Michigan Institutional Review Board (HUM00132484). All participants provided informed consent using the MacArthur Competence Assessment Tool for Clinical Research (MacCat-CR) (13) or the Evaluation to Sign an Informed Consent Document for Research (14).

Participants (male and female) between the ages of 12–45 were recruited for two arms of the study: cases (*N* = 25) and controls (*N* = 6). Inclusion criteria for the cases were as follows: (1) Minimal exposure to AP treatment, as defined as having previous AP exposure for equal to or less than 2 weeks within the past 3 months, and (2) Diagnostic and Statistical Manual of Mental Disorders-5 (DSM-5) or DSM-4 diagnosis of schizophrenia, schizoaffective disorder, schizophreniform disorder, delusional disorder, brief psychotic disorder, psychotic disorder not otherwise specified (NOS) or unspecified schizophrenia spectrum, major depressive disorder with psychotic symptoms, bipolar I disorder or bipolar II disorder with psychotic features. Psychiatric diagnosis was assessed at baseline by conducting either the Mini Neuropsychiatric Interview (MINI) for Psychotic Disorder Studies Version 7.0.2, Structured Clinical Interview for Axis I DSM-5 Disorders (SCID-5), or a medical chart review. Our diagnostic inclusion criteria were broad as our goal was to capture the metabolic effects of antipsychotics that occur early after medication exposure, and have been documented in multiple severe mental illnesses that are treated with APs (15). Furthermore, it can be challenging to recruit patients with psychotic features in early medication exposure studies if diagnostic criteria are too narrow, given that a diagnosis may not be clear early-on in the treatment of psychotic symptoms (16, 17). The approach of including patients based on symptoms and not specific diagnoses is also congruent with the Research domain criteria (RDoC) framework (18, 19).

Controls were included if they had an absence of current or past DSM-5 diagnosis other than a specific phobia or adjustment disorder. For both arms, exclusion criteria included clinically significant medical conditions (e.g., type 1 or 2 diabetes, kidney/liver disease, cancer, pregnancy), use of select groups of medications (e.g., treatment for lipids, glucose, or weight, anti-inflammatory medications, immunosuppressants), and moderate to severe substance use determined either through medical chart review or MINI.

Cases were treated naturalistically with APs by their psychiatrist and followed for 12 weeks. Baseline and endpoint (week 12) assessments were used for metabolomics assays. Controls were assessed at baseline only. Assessments for both groups included anthropometric measures (body weight, height, BMI, and waist circumference), blood pressure, and fasting laboratory measures (glucose, insulin, and lipid panel).

### 2.2. Metabolomic analysis

Metabolomic analysis of serum samples was conducted at the Michigan Comprehensive Regional Metabolomics Core at the University of Michigan. All samples were drawn in the morning, after an overnight fast, and after serum separation they were frozen on dry ice and stored in −80°F until time of sample transportation (on dry ice) and analysis. Samples did not undergo any freeze/thaw cycles prior to time of assay. Targeted, quantitative metabolomic analysis was performed for acylcarnitines, amino acids, free fatty acids, and bile acids after extraction and separation. In brief, the acylcarnitine assay involved separation by liquid chromatography and then measurement of metabolites using electrospray ionization and triple-quadrupole mass spectrometry, as described in more detail by Chace and colleagues (20). There were similarities in general separation approach and analysis method for the bile acids, as well with respect to separation by liquid chromatography and then analysis with triple-quadrupole mass spectrometry (21). The amino acids were analyzed with the Phenomenex EZfaast kit (Torrance, CA) by Metabolomics Core staff. Finally, for the free fatty acids analysis, after extraction (22), the lipid-rich fractions were analyzed for free fatty acids using Agilent 5,890 gas chromatograph with an Agilent HP 88 column (23).

### 2.3. Statistical analysis

Demographic and clinical data were compared using Fisher's exact test, *t-*tests, or ANOVA as appropriate using IBM Statistical Package for the Social Sciences (SPSS) Version 25. All statistical analyses of the metabolomic datasets were conducted using Metaboanalyst 5.0 (24). The quantified metabolomic datasets were transformed and scaled to achieve normal distribution for conducting parametric statistical analyses. *T*-tests were used to compare mean baseline metabolite concentrations between (1) AP-naïve cases and controls at baseline, and (2) AP-naïve cases who do and do not develop ≥5% body weight gain at 12 weeks. Finally, changes from baseline to endpoint between AP-naïve cases who did (i.e. ≥5%) and did not develop significant body weight gain were compared with a two-way repeated measures ANOVA test. Pearson correlations were calculated between change in weight and change in each metabolite concentrations from week 1 to week 12 for cases. To control for multiple comparisons, a false discovery rate (FDR) was calculated (25). Metabolites presenting with an FDR ≤ 0.05 were considered statistically significant. Fold-changes comparing metabolite mean concentrations were calculated to determine the direction of difference between groups of comparison for metabolites that met statistical significance criteria.

## 3. Results

Twenty-five minimally exposed AP cases were enrolled, and 17 cases completed both baseline and 12-week follow up visits, while the remaining 8 cases had baseline visits only. Six healthy controls completed baseline visits. Among cases, the most common diagnosis was unspecified schizophrenia spectrum and other psychotic disorder (*N* = 13). The mean duration of AP exposure at baseline was 7.9 days. Detailed participant demographics are available in Table 1.

**Table 1:** Baseline demographics of cases (*N* = 25) and controls (*N* = 6).

**Demographic**	Cases^*^	**Controls**	**P**	**Odds ratio**	**Cohen's d**
Age, Mean (SD)	21.8 (4.1)	25.2 (3.5)	0.069		−0.845
Sex, Female, *n*	8	3	0.638	0.663	
Race, *n*			0.536	5.519	
White	12	2			
Asian	6	4			
Black/African American	2	-			
Hispanic	1	-			
Middle Eastern	1	-			
Mixed	3	-			
Tobacco user, *n*	5	1	1.00	< 0.001	
Cannabis use, *n*	3	2	0.183	1.957	
**Metabolic measures, Mean (SD)**					
Body weight (kg)	70.8 (13.8)	64.5 (13.7)	0.342		0.458
BMI	24.7 (3.7)	22.6 (4.1)	0.291		0.555
Waist circumference (cm)	84.0 (9.5)	83.6 (12.6)	0.944		0.040
Fasting glucose (mmol/L)	5.1 (0.3)	5.1 (0.34)	0.812		−0.115
Fasting insulin (mmol/L)	8.7 (7.1)	5.2 (2.7)	0.070		0.541
HOMA-IR	2.0 (1.7)	1.2 (0.6)	0.076		0.520
Triglycerides (mmol/L)	0.8 (0.5)	0.9 (0.4)	0.968		−0.017
HDL cholesterol (mmol/L)	1.4 (0.5)	1.7 (0.5)	0.282		−0.527
LDL cholesterol (mmol/L)	2.1 (0.8)	2.8 (0.6)	0.026		−0.936
Total cholesterol (mmol/L)	3.9 (1.0)	4.9 (0.2)	< 0.001		−1.076
**Case diagnosis**, ***n***				
Unspecified schizophrenia spectrum and other psychotic disorder	14				
Bipolar I disorder	2				
Major depressive disorder with psychosis	5				
Schizophrenia	2				
Schizophreniform disorder	1				
Schizoaffective disorder	1				
**Case antipsychotic medication at baseline** ^**^				
**Low metabolic risk**, ***n***				
•Aripiprazole	10				
•Lurasidone	2				
**Moderate metabolic risk**, ***n***				
•Risperidone	7				
•Paliperidone	2				
•Quetiapine	2				
•Risperidone + Quetiapine	1				
**High metabolic risk**, ***n***				
Olanzapine	1				
Duration of antipsychotic exposure at baseline, Mean Days (SD)	8.0 (4.0)				

SD, standard deviation; BMI, body mass index; HOMA-IR, homeostasis model assessment of insulin resistance; HDL, high density lipoprotein; LDL, low density lipoprotein. Bolded indicate statistical significance (*p* < 0.05).

* Two study participants did not have lipid panel, glucose, insulin, or HOMA-IR data. Three participants did not have waist circumference or cannabis use data. Tobacco and cannabis use data were not available for one control participant.

** AAP risk groups were defined as follows: high = olanzapine or clozapine; moderate = risperidone, quetiapine, paliperidone, or iloperidone; low = aripiprazole, lurasidone, or ziprasidone. AP exposure duration is missing for one participant.

Following 12 weeks of AP exposure, cases experienced increases in body weight (*p* < 0.001), BMI (*p* < 0.001), waist circumference (*p* = 0.015), LDL cholesterol (*p* = 0.009), and total cholesterol (*p* = 0.027) (Table 2). Additionally, a subgroup of cases (*N* = 9) experienced clinically significant increases (≥5%) in body weight. The distribution of low and moderate metabolic risk medications was not significantly different (*p* = 0.453; data not shown) between the high and low weight gain groups (Table 2). There was also no significant difference between antipsychotic exposure prior to the baseline visit in the high and low weight gain groups (*p* = 0.330; data not shown).

**Table 2:** Changes in metabolic measures between baseline and week 12.

Metabolic measures, mean (SD)^*^	**All cases** (***N** =* **17)**			≥**5% weight gain (*****N** =* **9)**			<**5% weight gain (*****N** =* **8)**			**≥5 vs. < 5% Weight Gain**
	**Week 1**	**Week 12**	***P***	**Week 1**	**Week 12**	***P***	**Week 1**	**Week 12**	***P***	**Changes from week 1 to 12**, ***P***
Body weight (kg)	71.0 (11.6)	75.3 (12.6)	< 0.001	71.2 (14.3)	77.9 (15.1)	< 0.001	70.9 (8.4)	72.3 (9.2)	0.041	< 0.001
BMI	25.0 (3.1)	26.5 (3.5)	< 0.001	25.4 (3.9)	27.9 (4.0)	< 0.001	24.5 (2.0)	25.0 (2.3)	0.032	< 0.001
Waist circumference (cm)	83.2 (7.8)	86.6 (8.4)	0.015	83.2 (9.0)	89.4 (9.6)	0.004	83.1 (6.8)	83.5 (6.0)	0.765	0.016
Fasting glucose (mmol/L)	5.1 (0.3)	5.1 (0.5)	0.767	5.1 (0.4)	5.1 (0.6)	0.849	5.1 (0.2)	5.1 (0.3)	0.808	0.970
Fasting insulin (mmol/L)	8.5 (5.4)	10.1 (13.4)	0.476	7.9 (5.1)	7.6 (3.4)	0.859	9.1 (6.1)	14.4 (19.4)	0.459	0.402
HOMA-IR	1.9 (1.3)	2.4 (2.7)	0.525	1.8 (1.2)	1.7 (0.7)	0.843	2.1 (1.5)	3.1 (3.9)	0.489	0.431
TG (mmol/L)	0.9 (0.6)	1.1 (0.6)	0.256	0.7 (0.4)	0.9 (0.5)	0.225	1.2 (0.7)	1.3 (0.6)	0.620	0.866
HDL cholesterol (mmol/L)	1.4 (0.6)	1.3 (0.4)	0.294	1.6 (0.7)	1.4 (0.4)	0.277	1.3 (0.3)	1.3 (0.4)	0.926	0.299
LDL cholesterol (mmol/L)	2.2 (0.8)	2.6 (0.7)	0.009	2.1 (0.8)	2.3 (0.6)	0.179	2.4 (1.0)	3.0 (0.6)	0.027	0.092
Total cholesterol (mmol/L)	4.1 (1.0)	4.5 (0.8)	0.027	3.9 (1.2)	4.1 (0.7)	0.336	4.4 (1.0)	4.9 (0.8)	0.044	0.263

SD, standard deviation; BMI, body mass index; HOMA-IR, homeostatic model assessment of insulin resistance; TGs, triglycerides; HDL, high density lipoprotein; LDL, low density lipoprotein. Bolded indicate statistical significance (*p* < 0.05).

* Waist circumference, lipid panel, glucose, insulin, or HOMA-IR were not available for 2 participants (one each in ≥ and <5% weight gain groups). Differences between week 1 and 12 among all cases and within the ≥ and <5% weight gain groups were conducted using paired *t*-tests. Changes from week 1 to 12 were compared between the ≥ and <5% weight gain groups using repeated measures ANOVA.

### 3.1. Baseline serum metabolome features distinguishing cases from controls

Overall, 20 amino acids, 20 bile acids, 30 fatty acids, and 29 acylcarnitines were identified and quantified. The supplement contains lists of all metabolites identified in the metabolomics assays. Six fatty acids differentiated the two groups with an FDR of <0.05. Behenic acid (22:0), arachidonic acid [20:4 (n-6)], and myristoleic acid [14:1 (n-5)] were higher at baseline in cases, and palmitoleic acid [16:1 (n-7)t], lauric acid (12:0), and heneicosylic acid (21:0) were higher in controls at baseline. The Supplementary material also contains a table with FDR values for the six metabolites. No differences were observed for baseline levels of acylcarnitines, amino acids, nor bile acids between these two groups.

### 3.2. Metabolome features associated with variable weight gain (<5 vs. ≥5%) due to antipsychotics

**Figure 1:**
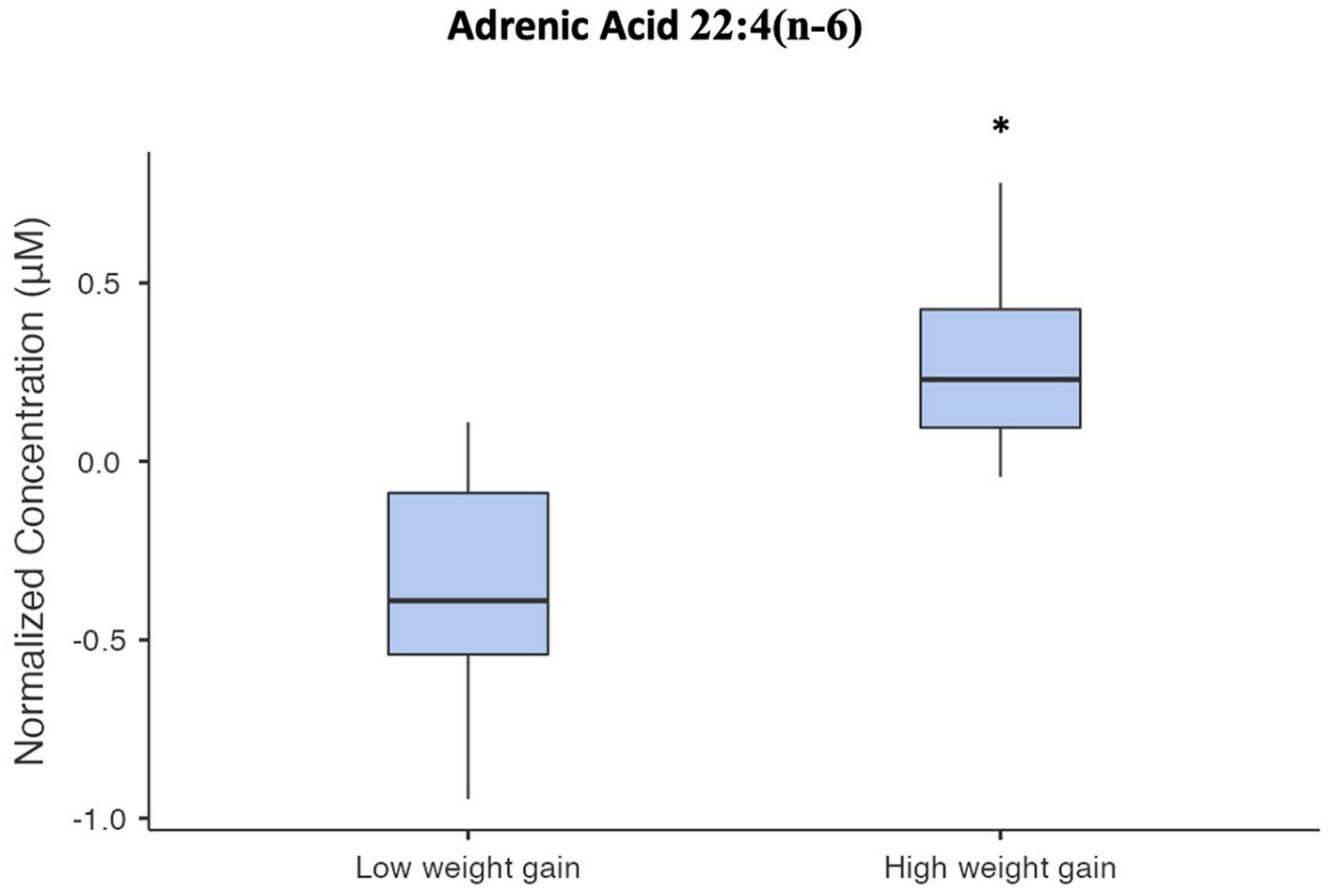
Box and whisker plot of normalized serum adrenic acid concentrations. The asterisk indicates that adrenic acid concentrations in the high-weight gain group were significantly different from the low-weight gain group (FDR = 0.0408).

Among the identified and quantified metabolites, none met the FDR threshold for significance as being altered from baseline to week 12 when changes were compared between case groups who gained <5 or ≥5% of their baseline body weight, or when examining associations between changes in metabolite concentrations and body weight over 12 weeks. Among metabolites differentiating these groups at baseline, baseline concentrations of the fatty acid adrenic acid [22:4(n-6)] were significantly elevated in cases who had more than 5% body weight gain compared to those who did not (FDR = 0.0408), as shown in Figure 1.

## 4. Discussion

Growing evidence supports metabolomic signatures as potential biomarkers for intrinsic and early AP-induced metabolic dysfunction in PSD. In this study, comparisons of metabolomic profiles of minimally AP exposed cases vs. healthy controls at baseline, identified six differential fatty acids. These include reduced palmitoleic acid, lauric acid, and henieocosylic acid levels, and elevated behenic acid, myristoleic acid, and arachidonic acid levels, of which increased arachidonic acid levels are consistent with a previous study (26). Palmitoleic acid and arachidonic acid are particularly interesting due to their roles in inflammation; inflammation has been proposed to underly intrinsic metabolic dysfunction in PSDs (27). Specifically, palmitoleic acid-rich supplementation has been shown to improve dyslipidemia through anti-inflammatory action (28–30). Alternatively, arachidonic acid is an omega-6 polyunsaturated fatty acid (PUFA); PUFAs, which are pro-inflammatory mediators. As such, lower circulating levels of palmitoleic acid and higher circulating levels of arachidonic acid may contribute to inflammation and hence intrinsic metabolic dysfunction in PSDs. Moreover, the conversion of arachidonic acid into inflammatory prostaglandins produces lipid peroxidation products, leading to oxidative stress (31), which in turn has been linked to metabolic dysfunction and PSD (32–35).

However, several association studies have illustrated that elevated circulating palmitoleic acid levels are found in individuals with obesity and metabolic syndrome (36, 37), and associate with higher triglyceride levels and insulin resistance in the general population (38, 39), contrary to the reduced palmitoleic acids observed in our study. Nonetheless, one study demonstrated that in individuals who are at risk for type 2 diabetes, reduced palmitoleic acid levels associate with greater insulin resistance (40). These findings perhaps suggest that the reduction in palmitoleic acid levels found in this study reflects the early, insulin resistant state commonly observed among AP-naïve first-episode psychosis patients (41). Given the small size of this pilot study, an interesting future direction would be to expand these results to a larger patient population to determine if reduced palmitoleic acid and increased arachidonic acid in serum may represent biomarkers of intrinsic metabolic dysfunction in PSD.

**Figure 2:**
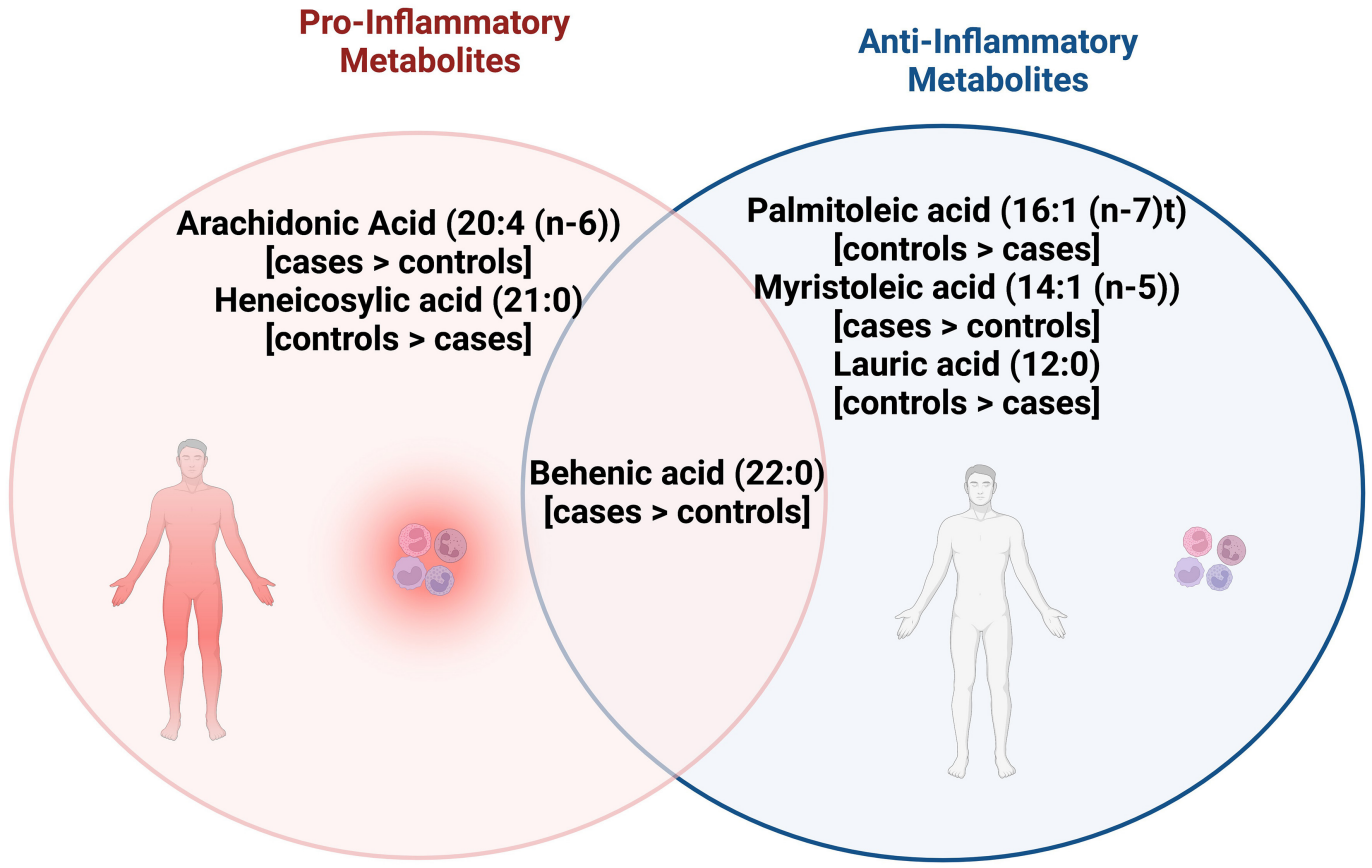
Venn diagram shows the pro and anti-inflammatory fatty acid metabolites that were significantly different between cases and controls, with behenic acid, arachidonic acid, and myristoleic acid observed to be higher in cases while palmitoleic acid, lauric acid, and heneicosylic acid were higher in controls. Behenic acid appears to have both pro and anti-inflammatory actions while arachidonic acid and heneicosylic acid have pro-inflammatory actions. Palmitoleic acid, myristoleic acid, lauric acid appear to have anti-inflammatory actions.

With respect to the other fatty acids identified as differentiating the cases and controls at baseline, behenic acid has been associated with anti-inflammatory activity in mice (42), and proinflammatory activity in humans (43). Heneicosylic acid appears to be minimally studied but may be inflammatory when considering it is a saturated fatty acid (44), whereas myristoleic acid and, conversely, the saturated lauric acid have been linked to anti-inflammatory activity (45–47). Figure 2 describes associations between fatty acids identified as significantly different between cases and controls in this pilot study, and whether published literature generally supports their role as inflammatory, or anti-inflammatory.

Following 12 weeks of AP exposure, cases had increased in body weight, BMI, waist circumference, total cholesterol, and LDL cholesterol. This increase in weight despite the fact that many of the cases were using antipsychotics with lower weight gain propensity is not necessarily surprising, given that research has suggested all antipsychotics cause weight gain in patients with minimal past exposure, but that the extent of weight gain is different between individual medications (3). Additionally, a subgroup developed clinically significant body weight gain (≥5%) and were distinguished at baseline by elevated levels of the fatty acid, adrenic acid. Studies have shown that specific triacylglycerols predict increases in body weight and BMI following AP treatment in first-episode psychosis patients (12). Our study extends these findings, suggesting elevation of adrenic acid as an additional novel early predictor of AP-induced weight gain. However, we were not able to replicate research from other teams with respect to the extent of changes in lipids; likely this was attributable to different testing platforms and identifiable metabolites, among other differences in study design, including length of medication exposure (48, 49). It is notable that elevations in adrenic acid have been associated with obesity (50), supporting its potential physiological relevance as a predictor of AP-induced weight gain. Adrenic acid is an omega-6 PUFA, which acts as pro-inflammatory mediators. Thus, it may contribute to inflammation, which may underly AP-induced metabolic dysfunction in patients with PSD (27). These findings may have important clinical implications to help identify individuals who are at high risk for AP-induced weight gain and may benefit from early prevention strategies. However, given the preliminary nature of this study, these results must be replicated in larger studies with longer duration of follow up.

Taken together, the prominence of fatty acid dysregulation in this study extends previous findings suggesting that dysregulated lipid metabolism may underlie psychosis illness and AP-induced metabolic dysfunction (12, 26). Our findings corroborate the growing body of evidence supporting individual variabilities in risk for AP-induced metabolic dysfunction and the importance of identifying early biomarkers that predict AP-induced weight gain.

While the physiological relevance of the identified biomarkers reinforces the clinical value of the findings, several limitations should be noted. First, relative to the case group, the control group had a small sample size, resulting in part from COVID-19 pandemic restrictions, where treatment studies with patients were allowed to continue, whereas healthy control studies were halted. The control group also had a larger percentage of patients who identified as Asian, as compared to the case group. These factors may have limited the statistical power in the analyses. Other potential confounders include physical activity levels and diet patterns, which were not considered in the present investigation. Furthermore, the heterogeneity of APs and diagnoses among cases may also have confounded the findings. While there is evidence demonstrating that the impact of antipsychotics on metabolic side effects is potentially disease agnostic (15), there are documented differences among antipsychotics in their ability to cause metabolic side effects (51, 52). This may, in part, explain why there were no detected changes in metabolites over 12 weeks of AP treatment associated with body weight change. Finally, the minimally exposed AP cases in the study had some AP exposure at baseline, which may have hindered examination of illness metabolomics independent of AP exposure. This may also have prevented investigation of the very early metabolic changes induced by APs, especially considering that changes in glucose metabolism can be induced by a single dose of APs (53). Nonetheless, there are ethical challenges to keeping patients AP-naïve for research purposes, which renders it difficult to impose too many exclusion criteria for an already difficult population to recruit. It is also possible that studies in patients with minimal, vs. absent, antipsychotic exposure may be more valuable to translating precision health research into practice given that it is unlikely antipsychotic treatment would be withheld to wait for biomarker results in a patient presenting acutely with psychosis.

## 5. Conclusion

Collectively, we demonstrate differences in fatty acid metabolites between controls and minimally exposed AP cases with PSD. Additionally, the fatty acid, adrenic acid, may represent an early predictor of AP-induced weight gain. Collectively, these candidate biomarkers provide mechanistic insight into intrinsic and AP-induced metabolic dysfunction in PSDs and represent potential targets for precision health approaches to mitigate metabolic dysfunction in PSDs. Future studies are needed with larger sample sizes and with cases who fully lack AP exposure to rigorously validate these findings before implementation in a clinical setting.

## Data availability statement

The original contributions presented in the study are included in the article/Supplementary material, further inquiries can be directed to the corresponding author.

## Ethics statement

The studies involving human participants were reviewed and approved by Research Ethics in Review Board of the Centre for Addiction and Mental Health (030/2017 and 060/2014) and the University of Michigan Institutional Review Board (HUM00132484). Written informed consent to participate in this study was provided by the participants' or legal guardian/next of kin.

## Author contributions

JL and KW performed the statistical and bioinformatics analyses. JL wrote the first draft of the manuscript. All authors contributed to study design and subsequent drafts.
