# Supplementary material for: Metabolomic signatures associated with weight gain and psychosis spectrum diagnoses: A pilot study
Source: Front Psychiatry. 2023 Apr 24;14:1169787. doi: 10.3389/fpsyt.2023.1169787 (PMC10164938; doi:10.3389/fpsyt.2023.1169787)
Supplement: Supplementary file 1 [file Table_1.DOCX]

Supplementary Material

Metabolomic signatures associated with weight gain and psychosis spectrum diagnoses: a pilot study

Jiwon Lee^1,2^, Kenya Costa-Dookhan^1,2^, Kristoffer Panganiban^1,2^, Nicole MacKenzie^1^, Quinn Casuccio Treen^1^, Araba Chintoh^1,2,3^, Gary Remington^1,2,3^, Daniel J. Müller^1,2,3,4^, Sanjeev Sockalingam^1,2,3^, Philip Gerretsen^1,2,3^, Marcos Sanches^1^, Alla Karnovsky^5^, Kathleen A. Stringer^6^, Vicki L. Ellingrod^6,7^, Ivy F. Tso^7,8^, Stephan F. Taylor^7^, Sri Mahavir Agarwal^1,2,3,9^, Margaret K. Hahn^1,2,3,9^†, Kristen M. Ward^5^†.

^1^Centre for Addiction and Mental Health, 250 College St, Toronto, Canada

^2^Institute of Medical Science, University of Toronto, 1 King’s College Circle, Toronto, Canada

^3^Department of Psychiatry, University of Toronto, 250 College Street, Toronto, Canada

^4^Department of Pharmacology and Toxicology, University of Toronto, 1 King’s College Circle, Toronto, Canada

^5^Department of Computational Medicine and Bioinformatics, University of Michigan Medical School, 100 Washtenaw Ave, Ann Arbor, MI, 48109

^6^Department of Clinical Pharmacy, University of Michigan College of Pharmacy, 428 Church Street, Ann Arbor, MI, 48109

^7^Department of Psychiatry, University of Michigan Medical School, 4250 Plymouth Road, Ann Arbor, MI, 48109

^8^Department of Psychiatry & Behavioral Health, Ohio State University, 520 King Ave, Columbus, OH, 43201

^9^Banting and Best Diabetes Centre, University of Toronto, 200 Elizabeth Street, Toronto, Canada

†**Equal contribution and senior authorship**: Margaret K. Hahn and Kristen M. Ward have shared senior authorship for this work

*** Correspondence:**Kristen M. Ward
[kmwiese@umich.edu](mailto:kmwiese@umich.edu)

# Supplementary Tables

**Supplementary Table 1.** List of identified metabolites in sample set

| **Acylcarnitines** | **Fatty Acids** | **Amino Acids** | **Bile Acids** |
| --- | --- | --- | --- |
| L-Car +Q1CC1:S1 | 12:0 | Phenylalanine | CDCA |
| C2 | 13:0 | Tryptophan | DCA |
| C3 | 14:0 | Leucine | UDCA/ HDCA |
| C5:0-DC | 14:1 (n-5) | Isoleucine | bMCA |
| C4 | 15:0 | Methionine | CA |
| C5 | 16:0 | Tyrosine | HCA |
| C6 | 16:1 (n-7)c | Proline | wMCA |
| C8:1 | 16:1 (n-7)t | Valine | GLCA |
| C8 | 18:0 | Alanine | GCDCA |
| C10:1 | 18:1(n-7) | Threonine | GDCA |
| C12-OH | 18:1(n-9) | Glutamic acid | GHDCA |
| C10 | 18:2 (n-6) | Glycine | GUDCA |
| C12:1 | 18:3 (n-3) | Aspartic acid | GCA |
| C14-OH | 18:3 (n-6) | Glutamine | TLCA |
| C12 | 19:0 | Serine | TCDCA |
| C14:1 | 20:0 | Asparagine | TDCA |
| C16-OH | 20:1 | Cystine | THDCA/ TUDCA |
| C14 | 20:2 | Histidine | TaMCA/ TbMCA |
| C18:2-OH | 20:3 (n-6) | Lysine | THCA |
| C16:1 | 20:4 (n-6) | Arginine | TCA |
| C20:4 | 20:5 (n-3) |  |  |
| C18:2 | 21:0 |  |  |
| C20:3 | 22:0 |  |  |
| C16 | 22:1 |  |  |
| C18:1 | 22:2 (n-6) |  |  |
| C20:2 | 22:4 (n-6) |  |  |
| C18 | 22:5 (n-3) |  |  |
| C20:1 | 22:6 (n-3) |  |  |
| C20:0 | 24:0 |  |  |
|  | 24:1 |  |  |

Supplementary Table 2. Fatty acid metabolites distinguishing antipsychotic-naïve cases (N=25) from controls (N=6) at baseline.

| **Fatty acid** | **P value** | **FDR** | **Log2(FC)** |
| --- | --- | --- | --- |
| Heneicosylic acid (21:0) | 0.000426 | 0.013204 | -1.5439 |
| Behenic acid (22:0) | 0.003219 | 0.043606 | 0.73381 |
| Palmitoleic acid (16:1 (n-7)t) | 0.005331 | 0.043606 | -1.7694 |
| Arachidonic acid (20:4 (n-6)) | 0.005627 | 0.043606 | 1.2012 |
| Myristoleic acid (14:1 (n-5)) | 0.008535 | 0.049883 | 0.69536 |
| Lauric acid (12:0) | 0.009655 | 0.049883 | -1 |
